# Supplementary material for: The Effect of Fiber Type and Yarn Diameter on Superhydrophobicity, Self-Cleaning Property, and Water Spray Resistance
Source: Polymers (Basel). 2021 Mar 7;13(5):817. doi: 10.3390/polym13050817 (PMC7962189; doi:10.3390/polym13050817)
Supplement: Supplementary file 1 [file polymers-13-00817-s001.pdf]

Supporting Information

The Effect of fiber type and yarn diameter on superhydrophobicity, self-cleaning property, and water spray resistance

Ji-Hyun Oh [1,2] and Chung Hee Park [1,*]

[1] Department of Textiles, Merchandising and Fashion Design, Seoul National University, Seoul, 08826, Republic of Korea; junghee@snu.ac.kr (C.H.P.)

[2] 2Department of Chemical and Biomolecular Engineering, North Carolina State University, Raleigh, North Carolina 27695, United States; joh25@ncsu.edu (J.H.O)

*Correspondence: junghee@snu.ac.kr

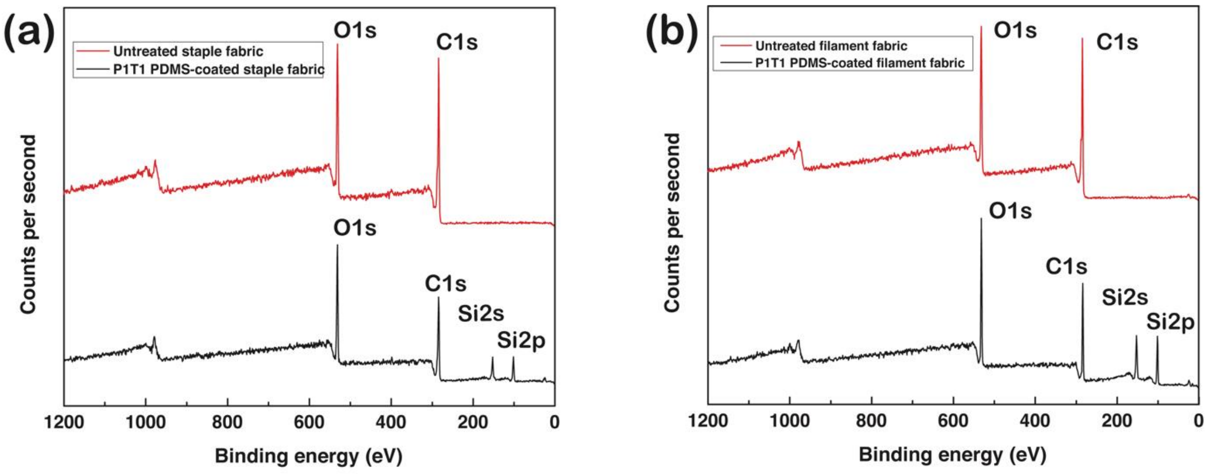

Figure 1. X-ray photoelectron spectroscopy (XPS) wide-scan of staple (a) and filament fabrics (b) before and after P1T1 PDMS-coating.

Table 1. Atomic concentration of elements detected from the X-ray photoelectron spectroscopy (XPS) of staple and filament fabrics before and after P1T1 PDMS-coating

	Atomic conc. [%]		
	C 1s	O 1s	Si 2p
Untreated staple fabric	75.1	24.9	
P1T1 PDMS-coated staple fabric	60.3	27.9	11.8
Untreated filament fabric	76.9	23.1	
P1T4 PDMS-coated filament fabric	50.7	28.1	21.3

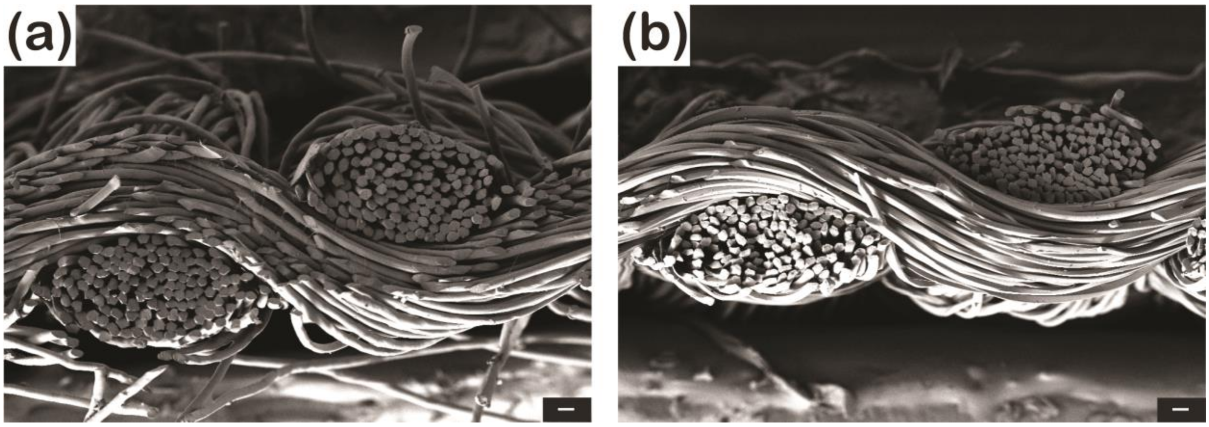

Figure 2. SEM images of cross section of staple fabric (a) and filament fabric (b).

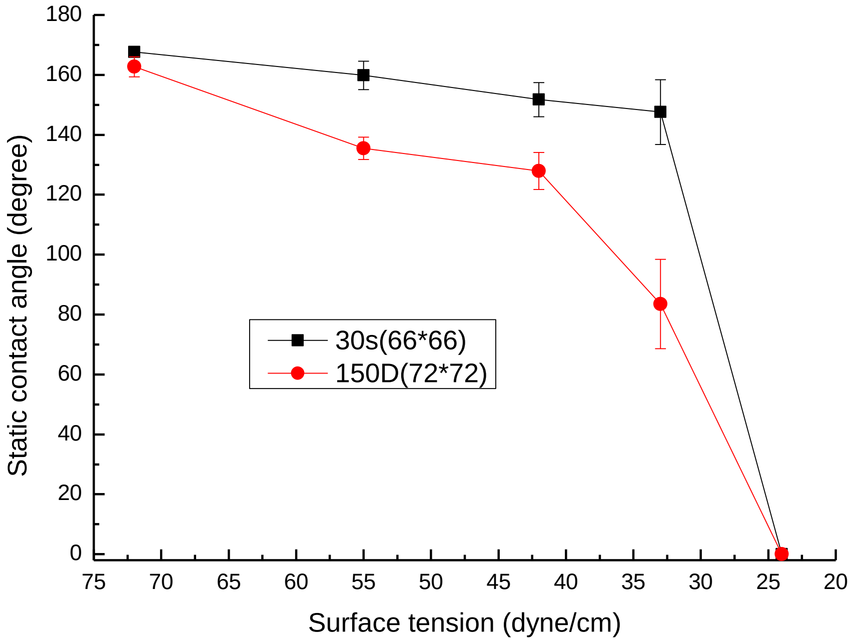

Figure 3. Static contact angles of P1T1 PDMS coated staple and filament fabrics depending on the surface tensions of droplets.

Table 2. Measurement of the rolling distance of water droplets on the staple fabrics contaminated without and with silicon carbides

*R.D.: Rolling Distance (cm), S.D.: Standard Deviation

	Untreated				P1T1 PDMS coating					
	w/o silicon carbides		w/ silicon carbides		w/o silicon carbides		w/ silicon carbides			
Tilted degree	10		10		10		10		15	
	R.D. (cm)	S.D.	R.D. (cm)	S.D.	R.D. (cm)	S.D.	R.D. (cm)	S.D.	R.D. (cm)	S.D.
Staple fabric	0	0	0	0	>4	0	0.1	0.17	>4	0

Table 3. Measurement of the rolling distance of water droplets on the filament fabrics contaminated without and with silicon carbides

*R.D.: Rolling Distance (cm), S.D.: Standard Deviation

	Untreated				P1T1 PDMS coating							
	w/o silicon carbides		w/ silicon carbides		w/o silicon carbides				w/ silicon carbides			
Tilted degree	10		10		10		15		10		15	
	R.D. (cm)	S.D.	R.D. (cm)	S.D.	R.D. (cm)	S.D.	R.D. (cm)	S.D.	R.D. (cm)	S.D.	R.D. (cm)	S.D.
Filament fabric	0	0	0	0	0.45	0.71	2.33	0.28	0.77	0.06	>4	0

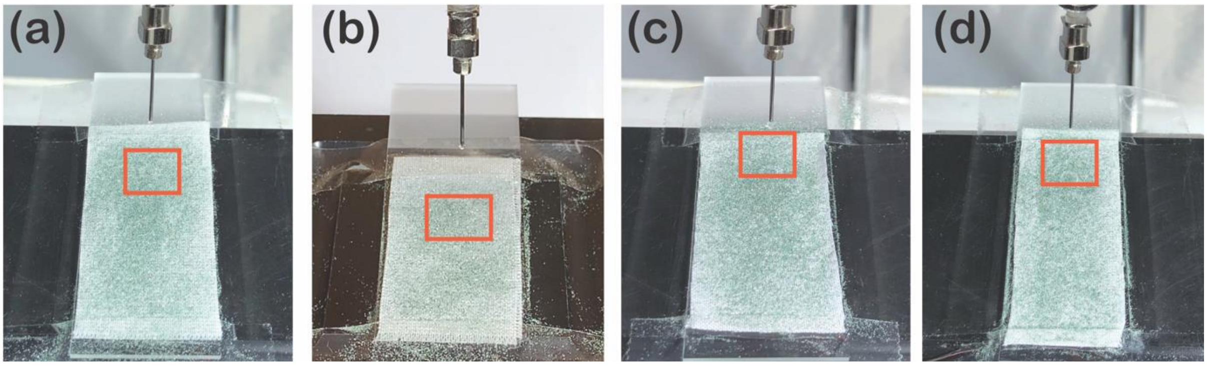

Figure 4. Photographs of self-cleaning property of untreated staple fabric tilted at 10° (a) and 15° (b) and untreated filament fabric tilted at 10° (c) and 15° (d). (Contaminant: silicon carbides).

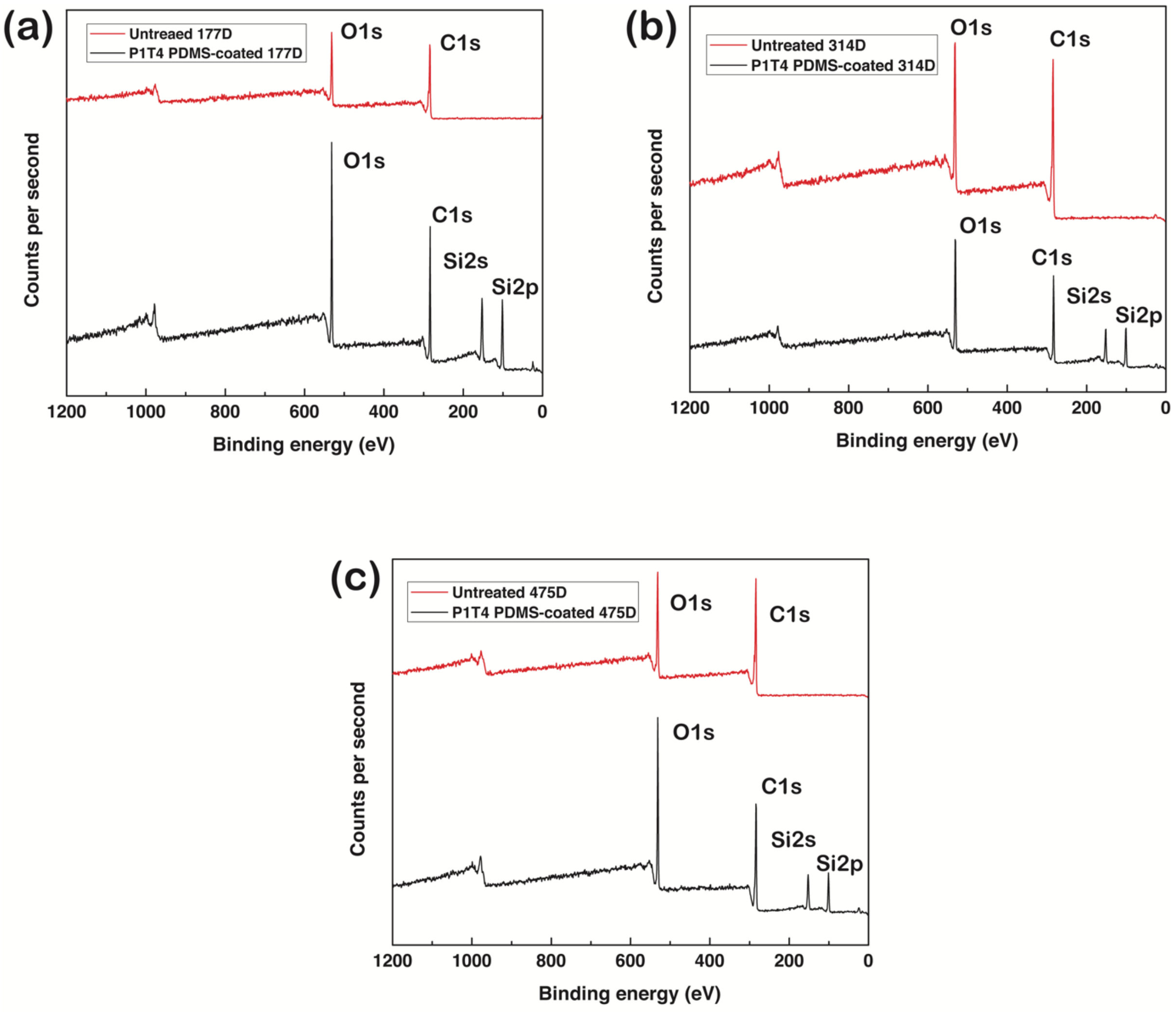

Figure 5. X-ray photoelectron spectroscopy (XPS) wide-scan of 177D (a), 314D (b) and 475D (c) before and after P1T4 PDMS-coating.

Table 4. Atomic concentration of elements detected from the X-ray photoelectron spectroscopy (XPS) of 177D, 314D and 475D before and after P1T4 PDMS-coating

	Atomic conc. [%]		
	C 1s	O 1s	Si 2p
Untreated 177D	76.2	23.8	
P1T4 PDMS-coated 177D	59.3	26.6	22.9
Untreated 315D	73.8	26.2	
P1T4 PDMS-coated 314D	50.1	27.0	19.9
Untreated 475D	76.5	23.5	
3P1T4 PDMS-coated 475D	51.7	28.4	14.1

Table 5. Atomic concentration of elements detected from the Energy-dispersive X-ray spectroscopy (EDS) of 177D, 314D and 475D before and after P1T4 PDMS-coating

	Atomic conc. [%]		
	C 1s	O 1s	Si 2p
untreated 177D	67.04	32.96	-
P1T1 PDMS-coated 177D	65.85	32.73	1.24
P1T4 PDMS-coated 177D	63.33	34.37	2.30
P1T7 PDMS-coated 177D	64.00	33.66	2.33
untreated 314D	64.57	34.43	-
P1T1 PDMS-coated 314D	67.40	31.04	1.56
P1T4 PDMS-coated 314D	63.10	34.94	1.96
P1T7 PDMS-coated 314D	67.65	29.42	2.93
untreated 475D	64.25	35.75	-
P1T1 PDMS-coated 475D	68.01	30.10	1.29
P1T4 PDMS-coated 475D	55.75	35.01	1.58
P1T7 PDMS-coated 475D	66.44	31.15	2.41

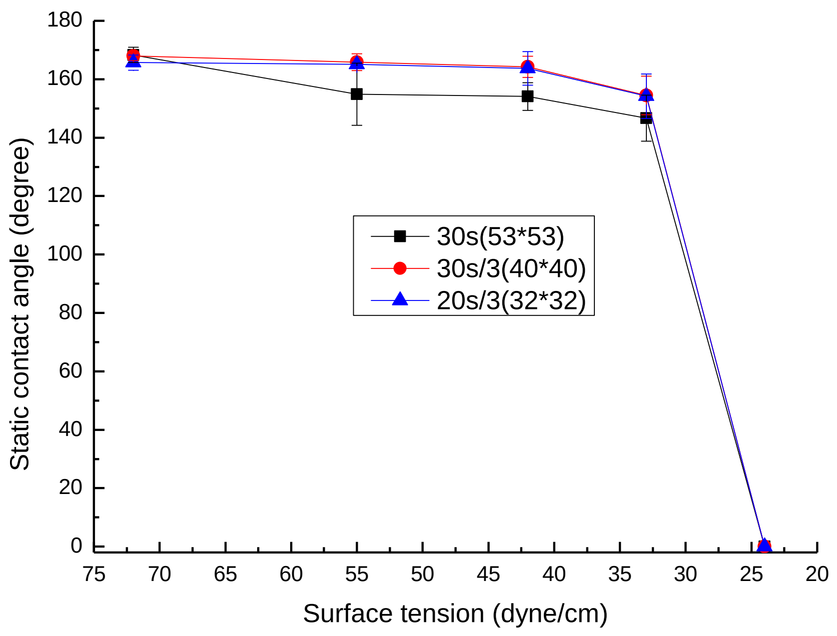

Figure 6. Static contact angles of 177D, 314D and 475D depending on the surface tensions of droplets.

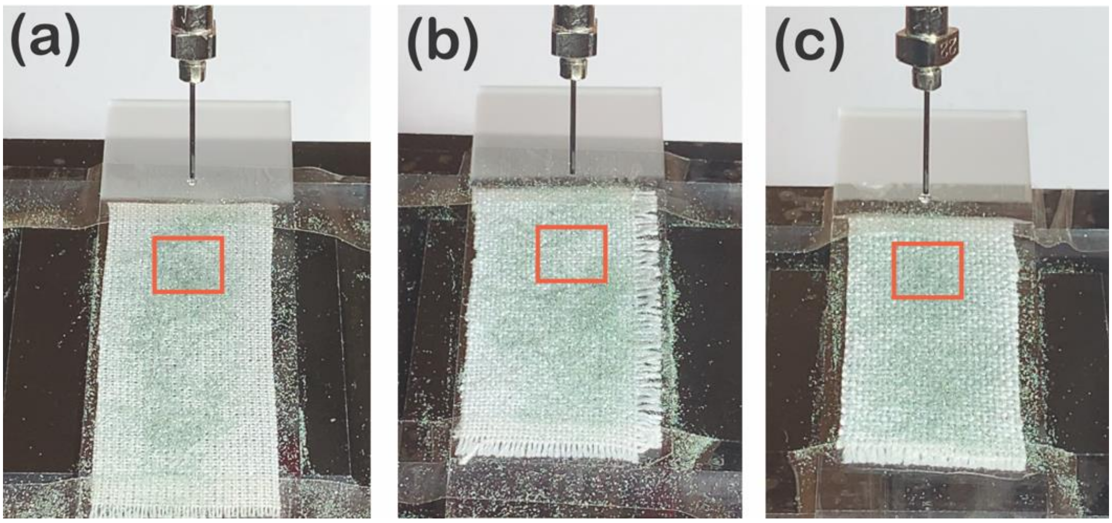

Figure 7. Photographs of self-cleaning property of untreated 177D (a), 314D (b) and 475D (c) tilted at 10°.

Table 6. Measurement of the rolling distance of water droplets on the 177D fabrics contaminated without and with silicon carbides

*R.D.: Rolling Distance (cm), S.D.: Standard Deviation

	Untreated				P1T4 PDMS coating							
	w/o silicon carbides		w/ silicon carbides		w/o silicon carbides		w/ silicon carbides					
Tilted degree	10		10		10		10		15		20	
	R.D. (cm)	S.D.	R.D. (cm)	S.D.	R.D. (cm)	S.D.	R.D. (cm)	S.D.	R.D. (cm)	S.D.	R.D. (cm)	S.D.
177D	0	0	0	0	>4	0	0.5	0.26	0.43	0.06	>4	0

Table 7. Measurement of the rolling distance of water droplets on the 314D fabrics contaminated without and with silicon carbides

*R.D.: Rolling Distance (cm), S.D.: Standard Deviation

	Untreated				P1T4 PDMS coating					
	w/o silicon carbides		w/ silicon carbides		w/o silicon carbides		w/ silicon carbides			
Tilted degree	10		10		10		10		15	
	R.D. (cm)	S.D.	R.D. (cm)	S.D.	R.D. (cm)	S.D.	R.D. (cm)	S.D.	R.D. (cm)	S.D.
314D	0	0	0	0	>4	0	0.37	0.35	>4	0

Table 8. Measurement of the rolling distance of water droplets on the 475D fabrics contaminated without and with silicon carbides

*R.D.: Rolling Distance (cm), S.D.: Standard Deviation

	Untreated				P1T4 PDMS coating							
	w/o silicon carbides		w/ silicon carbides		w/o silicon carbides				w/ silicon carbides			
Tilted degree	10		10		10		15		10		15	
	R.D. (cm)	S.D.	R.D. (cm)	S.D.	R.D. (cm)	S.D.	R.D. (cm)	S.D.	R.D. (cm)	S.D.	R.D. (cm)	S.D.
475D	0	0	0	0	1.17	0.15	>4	0	0	0	2.4	0.1
